# The Role of Transplacental Infection in *Leptospira* spp. Epidemiology in Cattle in Caatinga Biome, Brazil

**DOI:** 10.3390/microorganisms12061044

**Published:** 2024-05-22

**Authors:** Nathanael Natércio da Costa Barnabé, Rafael Rodrigues Soares, Deivyson Kelvis Silva Barros, João Pessoa Araújo Júnior, Camila Dantas Malossi, Maria Luana Cristiny Rodrigues Silva, Arthur Willian de Lima Brasil, Diego Figueiredo da Costa, Severino Silvano dos Santos Higino, Carolina de Sousa Américo Batista Santos, Sérgio Santos de Azevedo, Clebert José Alves

**Affiliations:** 1 Centro de Saúde Tecnologia Rural (CSTR), Universidade Federal de Campina Grande (UFCG), Av. Universitária, s/n, Santa Cecília, Patos 58708110, PB, Brazil; nathannaterciomv@gmail.com (N.N.d.C.B.); rafael_nh3@hotmail.com (R.R.S.); deivysonkelvis@hotmail.com (D.K.S.B.); luacristiny@yahoo.com.br (M.L.C.R.S.); severino.silvano@professor.ufcg.edu.br (S.S.d.S.H.); carolamerico@yahoo.com.br (C.d.S.A.B.S.); clebertja@uol.com.br (C.J.A.); 2 Instituto de Biociências, Departamento de Microbiologia e Imunologia, Universidade Estadual Paulista (Unesp), Av. Prof. Mário Rubens Guimarães Montenegro, s/n, Botucatu 18618687, SP, Brazil; joao.pessoa@unesp.br (J.P.A.J.); camilamalossi@gmail.com (C.D.M.); 3 Centro de Ciências Agrárias (CCA), Universidade Federal da Paraíba (UFPB), Rodovia BR 079, Km 02, Areia 58397000, PB, Brazil; arthurwillian7@yahoo.com.br (A.W.d.L.B.); diegoveter@hotmail.com (D.F.d.C.)

**Keywords:** *Leptospira* spp., semiarid, cows, embryos, fetuses, PCR

## Abstract

Leptospirosis is an infectious disease that affects domestic animals, wild animals, and humans. It represents a public health problem and has an important economic impact on livestock. This study aims to investigate the importance of genital and transplacental infection in the epidemiology of leptospirosis in cows maintained in Caatinga biome conditions, Northeastern Brazil, as well as reporting organs colonized by *Leptospira* spp. in embryos and fetuses. Blood, urinary tract (urine, bladder, and kidney), and reproductive tract (vaginal fluid, uterus, uterine tube, ovary, and placenta) samples were collected from 15 slaughtered pregnant cows. Two embryos and 13 fetuses were sampled. Central nervous system and choroid ovoid samples were collected from embryos. Blood, central nervous system, lung, peritoneal liquid, abomasal content, liver, spleen, urine, bladder, kidney, and reproductive system samples were collected from fetuses. Diagnostic methods included the microscopic agglutination test (MAT) using a collection of 24 serovars belonging to 17 different pathogenic serogroups of five species as antigens, as well as polymerase chain reaction (PCR). Anti-*Leptospira* spp. antibodies were found in 9 cows (60%), while 13 cows (86.67%) had at least one organ or urine with leptospiral DNA. No fetus was seroreactive. Among the embryos and fetuses, 13 (86.67%) presented leptospiral DNA, proving a high frequency of transplacental infection (100%). For cows, the most frequent biological materials regarding *Leptospira* spp. DNA detection were placenta (13 out of 15 samples; 86.7%), uterus (10 out of 15 samples; 66.7%), and vaginal fluid (5 out of 15 samples; 33.3%), while, for fetuses/embryos, the most frequent PCR-positive samples were choroid ovoid (1/2; 50%), spleen (6/13; 46.2%), kidney (5/13; 38.5%), and central nervous system (5/15; 33.3%). Sequenced samples based on the LipL32 gene presented 99% similarity with *L. borgpetersenii*. The results indicate that transplacental infection is an efficient way of spreading *Leptospira* spp. in cows maintained in Caatinga biome conditions. Therefore, prevention and control strategies must include actions that interrupt transmission through this alternative route.

## 1. Introduction

Leptospirosis is an infectious disease that affects domestic and wild animals worldwide [1]. Deemed a neglected re-emerging zoonosis, it represents a public health problem and has an important economic impact on livestock [2]. The etiological agent is pathogenic *Leptospira* spp., bacteria that are capable of rearranging their genome in the process of adaptation to new hosts, resulting in a complex epidemiological chain. This dynamism makes the prevention and control of leptospirosis challenging [3]. Exposure occurs through contact with infected animals or water and soil contaminated with urine [4]; in animals, transmission also occurs through contact with vaginal fluid and placental remains during mating [5]. In cattle, severe diseases are uncommon and are usually associated with infection by strains belonging to the Pomona, Icterohaemorrhagiae, and Grippotyphosa serogroups in young animals. Clinical signs and symptoms are rare, represented by pyrexia, hemolytic anemia, hemoglobinuria, jaundice, and, occasionally, meningitis [6].

As a consequence of the imbalance among human–animal–ecosystem interactions [7], it has been estimated that more than 1 million serious cases of human leptospirosis occur in the world annually, resulting in approximately 60 thousand deaths [8]. Referred to for decades as a tropical disease, the infection has raised concern in countries with temperate climates, where cases have increased due to the influence of global warming [9,10]. In livestock, losses result from abortions, weak offspring, retarded growth, low milk production or agalactia, and death [11]. In cattle, the main losses are associated with subfertility and early embryonic death [12].

Recent surveys have indicated a strong relationship between cases of leptospirosis in humans and bovine leptospirosis [13,14]—a warning to health authorities from Brazil, the country with the second largest cattle herd in the world (estimated at 202 million heads). Despite the unfavorable weather conditions for the survival of *Leptospira* spp., the agent has been shown to be common in cattle maintained in Caatinga biome conditions, an exclusively Brazilian biome, with emphasis on the genitals as an alternative route of transmission [15]. Sheep maintained under the same conditions showed a high frequency of transplacental infection [16]. However, there is a lack of research on the role of this alternative route for the spread of *Leptospira* spp. in cattle in the Caatinga biome.

The epidemiology of bovine leptospirosis is influenced by environmental factors, herd management practices, factors related to biosecurity, comorbidity with other infectious diseases, and individual factors such as age, sex, breed, and type of production [17]. In this scenario, the Caatinga is a biome with high solar radiation and long periods of water scarcity, in which it has stunted vegetation. The climate is semiarid, with rainfall concentrated in the summer/autumn between March and April. However, precipitation may occur between January and May. Droughts can last for more than a year, resulting in a negative water balance. Understanding the disease transmission chain in Caatinga biome cattle herds makes the improvement of surveillance, prevention, and control strategies possible, contributing to reducing the exposure of infection to other animals and humans. Therefore, this study aimed to investigate transplacental infection in the epidemiology of *Leptospira* spp. infection in cows maintained in Caatinga biome conditions in Brazil.

## 2. Material and Methods

### 2.1. Ethical Procedures

This survey was approved by the Animal Ethics Committee (CEUA) of the Federal University of Campina Grande, Brazil, protocol ID# 069-2018. 

### 2.2. Study Area

The public slaughterhouse of Patos county (latitude: 7°00′19″ S; longitude: 37°16′48″ W), Paraíba state, Northeast Region of Brazil, was used for biological sample collection during the period of September to November 2020. The animals belonged to properties covered by the Caatinga biome. The period during which this study was conducted corresponded to the dry season, with average precipitation and temperature of 0.47 mm and 29.28 °C, respectively [18]. 

### 2.3. Cows, Embryos, and Fetuses

The slaughter of production animals in early and mid-gestation stages of pregnancy is permitted by Brazilian legislation (https://www.planalto.gov.br/ccivil_03/_ato2015-2018/2017/decreto/d9013.htm (accessed on 12 April 2020)). Fifteen pregnant cows aged ≥24 months, cross-bred and with no history of vaccination against leptospirosis were used. Two embryos and 13 fetuses were collected. According to the data taken from animal movement forms held by the State Veterinary Service of Paraíba, these cows belonged to rural farms located in the municipalities of two federative units: Paraíba (counties of Condado, Olho D’água, Patos, Pombal, Santa Terezinha, São José de Espinharas, São José do Bonfim and São Mamede) and Pernambuco (counties of Buíque and Capoeiras).

### 2.4. Biological Sample Collection

Blood samples were collected from cows through puncture of the jugular vein using 8 mL labeled sterile tubes (Tantec, Glendale Heights, IL, USA) containing coagulation activator. Immediately after slaughter, urinary tract (urine, bladder, and kidney) and reproductive tract (vaginal fluid, uterus, uterine tube, ovary, and placenta) samples were collected, in addition to embryos and fetuses. At the laboratory, blood from the fetuses was collected through cardiac puncture using sterile tubes (8 mL). Blood samples were centrifuged at 1512× *g* for 10 min, and the serum samples were stored in microtubes (Eppendorf, São Paulo, SP, Brazil) at −20 °C for 48 h. 

Weight, diameter of choroid ovoid, and total length in the cranio-caudal direction were measured from the embryos; for the fetuses, weight, length between the base of the nape and the base of the tail, and the length between the withers and the distal far end of thoracic limb were measured to estimate age, using a methodology adapted from Barr et al. [19] and Tommasi Junior et al. [20]. Central nervous system, lung, liver, spleen, bladder, kidney, and reproductive system samples were collected; urine, peritoneal liquid, and abomasal content were obtained through direct puncture using sterile 1 mL syringes.

The tissue samples were immediately sectioned with autoclaved surgical materials and sterile blades for each tissue. After that, the tissues were deposited in autoclaved Petri dishes, avoiding contact between the fragments. Tissues were reduced to fragments of ≅ 1 g (in duplicate), placed into DNA- and RNA-free microtubes (Eppendorf, São Paulo, SP, Brazil), and stored at −20 °C for 48 h for molecular detection of *Leptospira* spp. Urine, peritoneal liquid, and abomasal content were also stored in duplicate in DNA- and RNA-free microtubes containing phosphate-buffered saline solution in a 1:1 ratio [21].

### 2.5. Polymerase Chain Reaction (PCR) and Sequencing

DNA was extracted from tissues, urine, peritoneal liquid, and abomasal content using the DNeasy Blood and Tissue Kit (Qiagen, Hilden, Germany), according to the manufacturer’s recommendations. The pathogenic leptospire-specific *LipL*32 gene was amplified with *LipL*32-45F (50′-AAG CAT TAC CGC TTG TGG TG-3′) and *LipL*32-286R (50′-GAA CTC CCA TTT CAG CGA TT-3′) primers [22], and PCR reactions were run based on previously described procedures [23]. PCR products were developed using 2% ultrapure agarose gel electrophoresis stained with Evans Blue (Thermo Fisher Scientific, Waltham, MA, USA) and 100 bp ladder, and DNA bands (242 bp) were visualized under ultraviolet light. Strain *Leptospira interrogans* serovar Copenhageni, Fiocruz L1-130 (ATCC BAA-1198) was used as the positive control, and ultrapure water was used as a negative control.

*LipL*32-45F and *LipL*32-286R primers [22] were used in the sequencing reactions with the Kit Big Dye Terminator v3.1 (Applied Biosystems, Foster City, CA, USA). A 3130xl Genetic Analyzer and POP-7 polymer [24] were used for capillary electrophoresis, sequence alignment was conducted by using BioEdit [25], and the data set strings were obtained from GenBank (National Biotechnology Information Center, Bethesda, MD, USA) using the BLAST tool. The SeaView4 software version 4 [26] was applied during the phylogenetic analysis, and the neighbor’s association model was used to build a phylogenetic tree with a bootstrap value of 1000 repetitions, as viewed through the FigTree v1.4.3 software. The phylogenetic reconstruction included *Leptospira* sequences for comparison.

### 2.6. Microscopic Agglutination Test (MAT)

Anti-*Leptospira* spp. antibodies were detected via the MAT technique using 24 serovars belonging to 17 different pathogenic serogroups of five species provided by the Laboratory of Veterinary Bacteriology of the “Universidade Federal Fluminense” (UFF), Niterói, Rio de Janeiro, Brazil as antigens, originating from the Pasteur Institute, France. The *Leptospira* species and serovars were *L. santarosai*: Shermani, Guaricura, and Canalzoni; *L. borgpetersenii*: Tarassovi, Ballum, Javanica, Castellonis, and Mini; *L. kirschneri*: Grippotyphosa and Cynopteri; *L. interrogans*: Autumnalis, Copenhageni, Canicola, Wolffi, Hardjoprajitno, Pomona, Icterohaemorrhagiae, Kennewicki, Pyrogenes, Hebdomadis, Bratislava, and Australis; and *L. noguchi*: Panamá and Lousiana [27]. Following the recommendation for diagnosing leptospirosis in cattle under Caatinga biome conditions, a cutoff point of 50 was deemed for cows [15], while a cutoff point of 25 was used for fetuses. Sera were sorted, the positive ones being double diluted, obtaining as a final result the highest titer achieved [27].

### 2.7. Data Analysis

The proportions of positive cows, embryos, fetuses, and biological samples were compared through the chi-squared test with Yates’ continuity correction or Fisher’s exact test using BioEstat 5.3 software [28], considering a 5% significance level (*p* ≤ 0.05).

## 3. Results

Most cows (9 of the 15 cows) were in the intermediate stage of pregnancy. Of the 15 embryos and fetuses, 7 were males and 5 were females. In three of them at a very premature stage, it was impossible to identify the sex or collect blood. Macroscopically, no lesions or abnormalities were observed.

Of the 15 cows, 9 presented antibodies, where the reactive serogroups were Sejroe, Tarassovi, and Australis. In particular, three animals were seropositive for Australis and Tarassovi at the minimum titer (cutoff 50), two animals seroreacted for Sejroe at titer 100, one animal was seroreactive for Sejroe at titer 400, one animal seroreacted for Sejroe at titer 800, and two animals were seroreactive for Sejroe and Tarassovi at titer 1600 (Table 1). No fetuses presented anti-*Leptospira* spp. antibodies (Table 2).

Leptospiral DNA was found in at least one sample in 13 cows (Table 1), as well as in their respective embryos and fetuses (*n* = 13; Table 2). Of the 120 cow samples, the molecular test detected *Leptospira* spp. DNA in 43. The most frequent biological materials regarding *Leptospira* spp. DNA detection were placenta (13 out of 15 samples), uterus (10 out of 15 samples), and vaginal fluid (5 out of 15 samples). There was no statistically significant difference (*p* > 0.05) between placenta and uterus; however, both biological materials were statistically different (*p* < 0.05) from the other ones (Table 3).

A total of 13 out of 15 embryos/fetuses presented leptospiral DNA: 1 embryo sample and 12 fetus samples (Table 2). Of the 131 samples, DNA was found in 35. The most frequent PCR-positive samples were choroid ovoid (1 out of 2 samples), spleen (6 out of 13 samples), kidney (5 out of 13 samples), and central nervous system (5 out of 15 samples). Spleen and kidney differed statistically (*p* < 0.05) from urine (Table 3).

**Figure 1:**
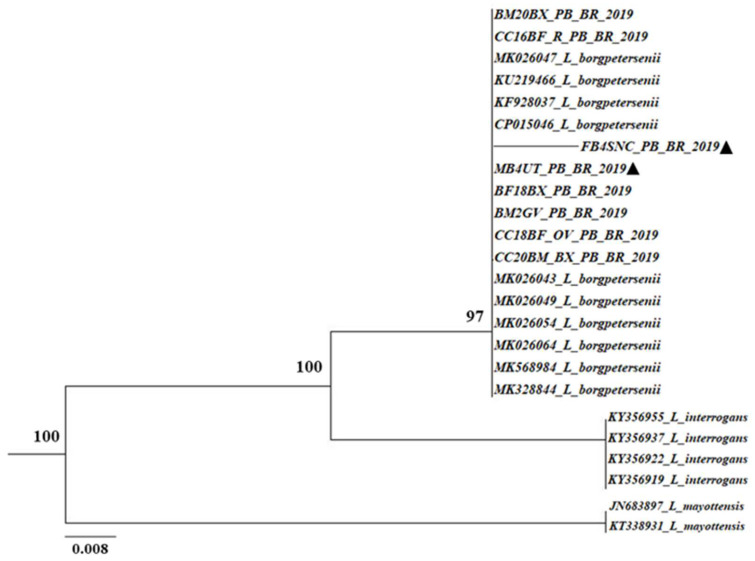
Phylogenetic tree based on the alignment of nucleotide sequences of the LipL32 gene *Leptospira* spp., constructed using the neighbor joining model with 1000 repetitions. Sequenced samples (▲).

Due to budget constraints, DNA sequencing from the PCR products was possible in only two samples (uterus and central nervous system of a cow and its fetus). These samples showed a 99% similarity with *Leptospira borgpetersenii* (Figure 1) strains R14-L chromosome 1, R6L chromosome 1, R14 chromosome 1, R6 chromosome 1, Mo4 chromosome 1, R23 chromosome 1, R28 chromosome 1, and R29 chromosome 1.

## 4. Discussion

The high frequency of seroreactivity found in cows indicates that, even under unfavorable climatic conditions, leptospirosis is widespread in cattle herds in the Caatinga biome. There was little variation in reactive serogroups (Sejroe, Tarassovi, and Australis) and, as has been reported in cattle worldwide [16,29], Sejroe was the most prevalent in this study. Previous research has reported the circulation of leptospires in cattle maintained in Caatinga biome conditions, with an average seropositivity of 52.38% and Sejroe and Tarassovi as the predominant serogroups [15]. 

The unfavorable climatic conditions (average rainfall and temperature of 0.47 mm and 29.28 °C, respectively, in the dry season) and a significant proportion of seroreactive cows—mainly for the Sejroe serogroup—provide evidence of intraspecies transmission, because cattle are deemed its natural hosts [30]. Tarassovi is one of the main serogroups found in bovine species [9], for which there have been few reports in Brazil [31]; however, it has been found in pigs [32] and “teiús” (*Tupinambis merianae*) [33] from the semiarid region. The Australis serogroup has pigs as reservoirs, but there are alternative wildlife hosts [30]; for example, equines act as their maintenance hosts in semiarid Brazil [34].

The serogroups detected here also infect humans, and Sejroe is among the most prevalent in human leptospirosis [13]. Emerging in New Zealand [13], Tarassovi predominates in reported cases of leptospirosis in dairy cattle workers. Australis is frequently detected in humans in Guatemala [35] and Colombia [36].

For cows, when comparing the positivity rates between different biological materials, there were statistically significant differences, demonstrating that strains adapted to cattle have tropism for placenta and uterus. The number of PCR-positive samples suggests that, when it is impossible to collect placenta and uterus, vaginal fluid can be used for the molecular diagnosis of leptospirosis. The greater frequency of micro-organism DNA in the reproductive tract compared to the urinary tract—especially in cases where only the reproductive tract was colonized—corroborates the occurrence of bovine genital leptospirosis [12], highlighting the agent–host adaptation process that enables transmission via alternative routes.

PCR proved that embryos and fetuses from positive cows were infected with *Leptospira* spp., showing a high frequency of transplacental infection (100%). Of the biological material of fetuses, the spleen, kidney, and central nervous system may reflect sites with suitable pH and availability of nutrients for the maintenance, growth, and multiplication of leptospires; however, leptospires were not detected in urine and presented low detection frequencies in peritoneal liquid, abomasal content, and reproductive tract. Furthermore, of the four lowest frequencies of PCR-positive samples, three corresponded to liquids (peritoneal liquid, abomasal content, and urine). This explains the statistical differences between the spleen and urine and kidney and urine, in relation to positivity. Although the investigation of multiple organs is ideal for the molecular diagnosis of leptospirosis in bovine fetuses [37], our results indicate that, if it is impossible to collect many samples, the spleen and kidney may be the biological material of choice.

For many decades, *Leptospira* spp. bovine genital tract infection has been considered a secondary effect of renal infection, as a result of bacteremia; however, there is evidence in the literature suggesting that genital leptospirosis should be considered a specific syndrome dissociated from renal/systemic disease, namely, Genital Bovine Leptospirosis [12]. In this regard, it is reasonable to suggest that the infection of fetuses/embryos in this study was due to the presence of leptospires in the genital tract of cows, taking into consideration that 13 cows and 13 fetuses/embryos presented *Leptospira* spp. DNA in at least one biological sample. Thus, Genital Bovine Leptospirosis may be an important syndrome in the Caatinga biome conditions. 

The sequenced DNA from two samples demonstrated 99% similarity with *L. borgpetersenii*—which belongs to the pathogenic clade and, according to virulence, the subgroup 2, along with the species *L. santarosai*, *L. mayottensis*, *L. weilii*, and *L. alexanderi* [38]. Adapted to cattle, *L. borgpetersenii* has the smallest genome compared to other pathogenic and saprobic strains, which determines its low resistance to the environment [39]. It causes early embryonic loss and estrus repetition, resulting from uterine inflammation and damage due to embryo invasion [12]. The identification of this *Leptospira* spp. species in the uterus of cows and in the central nervous system of fetuses suggests transplacental infection. This alternative route—as well as the venereal route [18,19]—may be responsible for the spread of these bacteria in cattle during the dry period in the Caatinga biome. In addition, *L. borgpetersenii* commonly infects humans [40].

Fetuses with low amounts of *Leptospira* spp. DNA and cows with higher antibody titers suggest humoral protection and reinforce the concept that the vaccination of cows can protect the conceptus, as well as contributing to attenuation of the strain’s virulence in the phase of increasing production of immunoglobulins subsequent to leptospiremia and intercurrent to leptospiruria. Our findings regarding the agent’s DNA detected in the reproductive tract of these cows highlight the importance of the genital carrier in the epidemiology of the disease. The circumstances of embryo/fetuses with many PCR-positive samples from cows with low antibody titers suggest the insufficient level of maternal antibodies for protection, or the stage of infection after the systemic condition, based on number of positive cows in the urinary and reproductive tracts. Embryo #6 was free of leptospire infection, as was the cow, even though it tested positive for Tarassovi (titer 50). This indicates that, at some point, there was contact between cow and pathogen. Fetuses with disseminated infection from seronegative cows demonstrate a lack of maternal humoral protection; this scenario may characterize leptospiremia, especially in the period preceding the production of antigens (immunological window). Other cows in the same situation presented leptospiral DNA in urinary and reproductive tracts, revealing systemic infection. A significant proportion of cows (38.46%) were positive only in the reproductive tract.

In the Caatinga biome, where climatic conditions are unfavorable for the survival of *Leptospira* spp., transplacental infection is an efficient alternative route that is less dependent on the environment. Therefore, in the dry season, the urinary route may be less relevant to the spread of the micro-organism. Given this information, measures to prevent and control bovine leptospirosis must include actions aimed at genital leptospirosis, as transplacental infection occurs when uterine immunity decreases, and the conceptus is invaded by leptospires present in the genital tract [30]. Vaccines licensed for animal use are formulations based on inactivated leptospires and, although they prevent clinical manifestations, reinfection, and simultaneous colonization of organs, they do not confer sterilizing immunity [1]; thus, the carrier animal should be treated. The vaccination protocol recommends immunizing heifers every four months, with the last dose being administered before the breeding season, aiming to ensure a maximum antibody response during conception and early to mid-gestation [41]. Calves should receive the first dose in the second month of life, with a booster from 21 to 28 days, and annual re-vaccination [42]. The recommended therapeutic protocol for ruminants—a single dose of streptomycin (25 mg/kg, IM)—is ineffective for the treatment of genital tract infection. However, when treatment was extended to three consecutive days, at the same dose, the drug was 94.1% effective in eliminating genital carrier status in naturally infected cows [43].

Isolation of leptospires from biological sample cultures is the gold standard for diagnosis; however, this methodology was not used in the survey, which is a limitation as molecular characterization of isolates is important for epidemiologic studies of strains infecting animals from a given region. However, leptospire isolation is very laborious, can take up to three months, and may present false-negative results. Moreover, PCR is a reliable, fast, and high-sensitivity technique, which is considered useful for the detection of *Leptospira* spp. carrier animals [5]. 

## 5. Conclusions

The obtained results indicate that *Leptospira* spp. efficiently spread via transplacental infection in cows maintained in Caatinga biome conditions in Northeastern Brazil. Therefore, prevention and control strategies for this disease must include actions that interrupt transmission through this alternative route, which is important to avoid the spread of leptospires through the animal–environment–human interface.

## Figures and Tables

**Table 1:** Serological and molecular detection of *Leptospira* spp. antibodies and DNA in cows from the Caatinga biome, Brazil.

Animal	MAT	PCR							
	9/15 (60.00%)								
	Serogroup (Titer)	13/15 (86.67%)							
		Urinary Tract			Reproductive Tract				
		Urine	Bladder	Kidney	Vaginal Fluid	Uterus	Uterine Tube	Ovary	Placenta
1	Sejroe (100)	+	−	+	+	+	−	−	+
2	Sejroe (1600)	−	−	−	+	+	−	−	+
3	Sejroe (100)	−	+	−	+	+	−	−	+
4	-	−	−	+	+	▲	−	+	+
5	Australis (50)	−	+	−	−	−	+	−	+
6	Tarassovi (50)	−	−	−	−	−	−	−	−
7	Sejroe (800)	−	−	+	−	−	−	−	+
8	Tarassovi (1600)	−	−	−	−	−	−	−	−
9	Tarassovi (50)	−	−	+	−	+	−	−	+
10	-	−	−	−	−	−	−	−	+
11	-	−	−	−	−	+	−	−	+
12	-	−	−	−	−	+	+	−	+
13	-	−	+	−	+	+	+	−	+
14	-	−	+	−	−	+	−	+	+
15	Sejroe (400)	−	−	−	−	+	+	−	+

+ = positive samples; − = negative samples; ▲ = leptospiral DNA sequenced.

**Table 2:** Serological and molecular detection of *Leptospira* spp. antibodies and DNA in cattle embryos and fetuses from the Caatinga biome.

Embryo/ Fetus	Sex	Dimension (cm)	Weight (kg)	Age	MAT	PCR										
						Central Nervous System	Choroid Ovoid	Lung	Peritoneal Liquid	Abomasal Content	Liver	Spleen	Urine	Bladder	Kidney	Reproductive System
1	F	19 × 27	0.531	4 months	−	+	f	−	−	−	+	−	⊅	−	+	−
2	M	20 × 32	1.015	5 months	−	−	f	−	−	−	+	−	−	−	−	−
3	◊	04 × 09	0.040	<3 months	⊄	−	f	+	−	−	−	−	−	−	−	−
4	M	22 × 33	1.291	5 months	−	▲	f	+	+	−	−	+	−	−	−	+
5	F	33 × 51	4.554	6 months	−	+	f	−	−	−	−	−	−	+	+	−
6•	◊	02 × 05	0.005	<2 months	⊄	−	−	ɇ	ɇ	ɇ	ɇ	ɇ	ɇ	ɇ	ɇ	ɇ
7	F	12 × 23	1.400	4 months	−	−	f	−	+	−	−	+	−	+	+	−
8	M	42 × 63	5.510	7 months	−	−	f	−	−	−	−	−	−	−	−	−
9•	◊	01 × 02	0.001	1 month	⊄	+	+	ɇ	ɇ	ɇ	ɇ	ɇ	ɇ	ɇ	ɇ	ɇ
10	F	08 × 16	0.113	3 months	−	−	f	−	−	+	−	+	−	−	−	−
11	M	29 × 48	2.911	6 months	−	−	f	+	+	+	+	+	−	+	+	−
12	F	50 × 69	8.227	8 months	−	+	f	−	−	−	+	−	⊅	−	−	−
13	M	20 × 32	0.948	5 months	−	−	f	−	−	−	−	+	−	−	−	−
14	M	15 × 24	0.468	4 months	−	−	f	−	−	−	−	+	⊅	+	+	−
15	M	15 × 26	0.502	4 months	−	−	f	+	−	−	−	−	−	−	−	−

• = embryo; F = female; M = male; ◊ = undefined sex; dimension = height × length; + = positive samples; − = negative samples; f = embryos-restricted structure; ɇ = fetuses-restricted biological material; ⊄ = could not collect; ⊅ = did not contain; and ▲ = leptospiral DNA sequenced.

**Table 3:** Molecular detection of *Leptospira* spp. DNA according to the biological material from cows, embryos, and fetuses.

Cows		Embryos and Fetuses	
Biological Material	Positive Samples/Total of Samples (%)	Biological Material	Positive Samples/Total of Samples (%)
Placenta	13/15 (86.67) ^a^	Choroid ovoid	1/2 (50.00)
Uterus	10/15 (66.67) ^a^	Spleen	6/13 (46.15) ^a^
Vaginal fluid	5/15 (33.33) ^b^	Kidney	5/13 (38.46) ^a^
Bladder	4/15 (26.67) ^b^	Central nervous system	5/15 (33.33) ^ab^
Kidney	4/15 (26.67) ^b^	Lung	4/13 (30.77) ^ab^
Uterine tube	4/15 (26.67) ^b^	Liver	4/13 (30.77) ^ab^
Ovary	2/15 (13.33) ^b^	Bladder	4/13 (30.77) ^ab^
Urine	1/15 (6.67) ^b^	Peritoneal liquid	3/13 (23.08) ^ab^
-	-	Abomasal content	2/13 (15.38) ^ab^
-	-	Reproductive system	1/13 (07.69) ^ab^
-	-	Urine	0/10 (00.00) ^b^
Total	43/120 (35.83)	Total	35/131 (26.72)

(%) = percentage of positive samples; different lowercase letters in the same column indicate significantly different proportions (*p* ≤ 0.05). Absence of letter = not included in statistical analysis.

## Data Availability

Dataset available on request from the authors.
